# A Case of Ruptured Splenic Artery Aneurysm in Pregnancy

**DOI:** 10.1155/2014/793735

**Published:** 2014-12-09

**Authors:** Elizabeth K. Corey, Scott A. Harvey, Lynnae M. Sauvage, Justin C. Bohrer

**Affiliations:** ^1^Department of Obstetrics and Gynecology, University of Wisconsin, School of Medicine and Public Health, Madison, WI 53715, USA; ^2^Department of Obstetrics, Gynecology, and Women's Health, University of Hawai‘i, John A. Burns School of Medicine, Honolulu, HI 96813, USA

## Abstract

*Background*. Rupture of a splenic artery aneurysm is rare complication of pregnancy that is associated with a significant maternal and fetal mortality. *Case*. A multiparous female presented in the third trimester with hypotension, tachycardia, and altered mental status. A ruptured splenic artery aneurysm was discovered at the time of laparotomy and cesarean delivery. The patient made a full recovery following resection of the aneurysm. The neonate survived but suffered severe neurologic impairment. *Conclusion*. The diagnosis of ruptured splenic artery aneurysm should be considered in a pregnant woman presenting with signs of intra-abdominal hemorrhage. Early intervention by a multidisciplinary surgical team is key to preserving the life of the mother and fetus.

## 1. Introduction

Rupture of a splenic artery aneurysm (SAA) is a rare condition that occurs predominantly in pregnancy. It is associated with a maternal mortality rate of 75% and fetal mortality rate of 95% [1]. We report a case of ruptured splenic artery aneurysm during the third trimester of pregnancy with both maternal and fetal survival.

## 2. Case Presentation

A 29-year-old multiparous female at 35 weeks and 6 days of gestational age arrived at our obstetrical triage unit in extremis with the chief complaint of abdominal pain. She was transported to the hospital by her male partner who provided the history. The abdominal pain was of abrupt onset approximately 1.5 hours prior to arrival and was mainly epigastric in origin. The partner observed a single episode of emesis en route and the gradual onset of confusion. The patient's prenatal course had otherwise been unremarkable. A physical examination revealed a lethargic and confused gravid female in apparent distress. The fundal height was 35 cm and the abdomen was diffusely tender. There was evidence of involuntary guarding and an absence of rebound tenderness. The systolic blood pressure was 60 mmHg as determined by palpation, pulse was 150 beats per minute, and body temperature was 38.2°C. She was able to localize pain and respond to simple commands. The obstetric history was significant for two prior uncomplicated spontaneous vaginal deliveries, one at term and one late preterm. An ultrasound study of the abdomen was performed at the bedside and showed a singleton fetus with a fetal heart rate of 70 beats per minute and free peritoneal fluid. The cervix was 3 cm dilated by digital examination, the membranes were intact, and there was no evidence of significant vaginal bleeding.

Two large bore intravenous lines were placed and aggressive fluid replacement was undertaken with normal saline. The systolic blood pressure improved modestly to 70/30. The fetal bradycardia persisted despite aggressive volume replacement, oxygen supplementation via nasal cannula, and position changes. An emergent exploratory laparotomy with cesarean delivery under general anesthesia was performed for the indications of suspected intra-abdominal hemorrhage and nonreassuring fetal heart tones. At the time of initial evaluation, the most likely cause of the patient's symptoms was assumed to be uterine rupture. A Pfannenstiel incision was used to access the abdomen. Upon entering the peritoneal cavity, 1500 mL of hemoperitoneum was encountered. A viable female infant was delivered through a low transverse hysterotomy and transferred to the neonatologist for resuscitation. The hysterotomy site was closed and the abdomen and pelvis were packed with laparotomy sponges to control the hemorrhage and identify the source of bleeding. The packs were removed from the pelvis in a stepwise fashion. The uterus and accompanying pelvic structures were intact and were not the source of hemorrhage. Upon removing the packs from the upper abdomen, brisk bleeding was encountered following removal of the packs in the left upper quadrant. An intraoperative consultation was obtained from a general surgeon who created a midline vertical incision extending to the xiphoid. The lesser sac was opened revealing copious blood clot and a peripancreatic hematoma. Heavy bleeding from this area was encountered stemming from the spleen. A splenectomy and distal pancreatectomy were performed with cessation of bleeding. Intraoperatively, she received six units of packed red blood cells, four liters of crystallized fluids, four units of fresh frozen plasma, and one pack of pooled platelets after an estimated three-liter blood loss. She received two prophylactic doses of Cefazolin. Her abdomen was closed after a Jackson Pratt drain was placed, and she was transferred to the Intensive Care Unit.

Postoperatively, broad spectrum antibiotics were administered empirically for 6 days for leukocytosis and persistent fever to 38.2°C. A discrete source of infection was not identified. Appropriate vaccinations were administered to* Haemophilus* influenza, seasonal influenza, pneumococcus, and meningococcus. She was discharged home in stable condition on hospital day 8 with one week of oral antibiotics. The patient's long-term course was complicated by a pancreatic fistula that resolved with conservative management. Final pathology revealed a 165-gram spleen measuring 10.8 × 6.8 × 4.5 cm with a large aneurysm of the main splenic arterial branch.

The neonate was born with APGARS 0 and 2, an arterial blood gas pH of 6.507, pCO_2_ of 176, and a base excess of −29.4. The neonate was resuscitated, intubated, and given head cooling measures for suspected hypoxic ischemic encephalopathy. An MRI of the head showed multiple brainstem infarcts. The neonate had multiple procedures performed to include tracheostomy and percutaneous gastrostomy. The infant was with severe neurologic impairment at one year of age.

## 3. Comment

**Figure 1:**
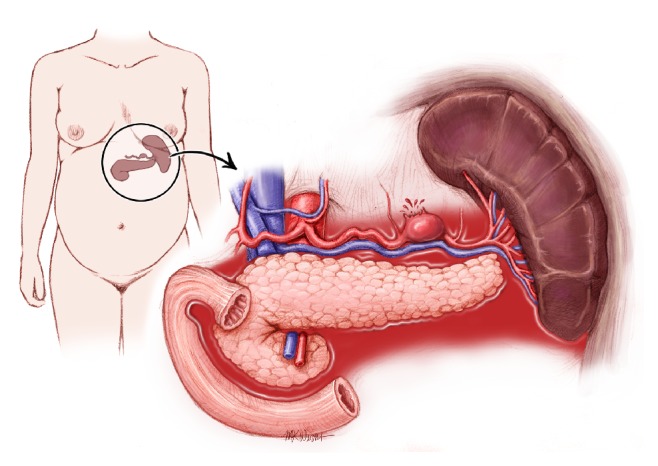
Rupture of splenic artery aneurysm during pregnancy.

Splenic artery aneurysm is the most common of all the visceral artery aneurysms [1]. SAA is defined by a pathologic dilatation of the splenic artery to greater than 1 cm in diameter (Figure 1) [2]. The prevalence in the general population is thought to be low at around 0.1-0.2%, although an autopsy study in patients 60 years of age or older found a prevalence as high as 10.4% [3]. The true prevalence remains unknown because 95% of individuals remain asymptomatic until the aneurysm ruptures [4–6].

SAA is an uncommon condition that occurs four times more frequently in women compared to men [2, 3]. Other risk factors include portal hypertension, congenital abnormalities of the vessels, inherited vascular and connective tissue disorders, vascular trauma, inflammatory processes, and degenerative arterial disease [2]. SAA is associated with pregnancy and the risk increases with increasing parity [1]. Hormonal and physiologic changes have been proposed to explain the increased incidence of SAA in pregnancy [7]. Estrogen, progesterone, and relaxin are all thought to play a role in the histologic remodeling of the arterial wall, predisposing to aneurysm formation through a process of medial degeneration and fragmentation of the elastic fibers [6]. Physiologic changes during pregnancy that can lead to SAA include increased cardiac output and blood volume [1]. These changes lead to increased blood flow, portal hypertension, and splenic arteriovenous shunting [2]. The increased splanchnic and splenic arterial blood flow may cause increased mechanical resistance on vessel walls leading to weakening [6].

The physical signs of a rupture most often include sudden and intense abdominal pain, most commonly in the left upper quadrant or epigastrium. The pain is frequently described as sharp and can radiate to the top of the left shoulder (Kehr's sign) [2]. Nausea and vomiting can also accompany the pain [1]. Hemorrhagic shock and circulatory collapse may occur within several minutes [6]. In the majority of cases, the rupture progresses rapidly, while, in 25% of cases, it occurs in two stages [1]. In a two-stage model of rupture, the initial hemorrhage is limited when blood clots block the foramen of Winslow, temporarily containing the hemorrhage to the lesser sac [2]. This stage may be accompanied by mild to moderate pain and can last hours to days. The second stage is characterized by free rupture through the foramen of Winslow into the abdominal cavity, which can lead to sudden maternal and fetal death [6]. The two-stage rupture can allow time for effective diagnosis and treatment to occur between the initial symptoms and the free rupture into the abdominal cavity [4].

Approximately, 95% of SAA rupture occurs during pregnancy, most commonly during the third trimester [8]. If a woman has an existing SAA, the risk of rupture during pregnancy is 20–50%. Though the rupture of a SAA during pregnancy is a rare event, it carries a high risk of maternal and fetal mortality. The mortality in the general population when a SAA ruptures is 25%. In pregnant women, this rate increases to a 75% maternal mortality rate and a 95% fetal mortality rate [1].

SAA rupture in pregnancy is difficult to diagnose since its symptoms mimic other obstetric and surgical emergencies. Approximately, 70% of cases of SAA rupture during pregnancy are misdiagnosed as uterine rupture [4]. Other common misdiagnoses include placental abruption, amniotic fluid embolism, or perforated peptic ulcer [5]. During pregnancy, diagnosis of both an unruptured and ruptured SAA is best performed by ultrasound with pulsed Doppler [1]. In the setting of a ruptured SAA, ultrasound will show free fluid in the abdomen, and the diagnosis is confirmed at the time of emergent laparotomy [1].

During a suspected case of SAA rupture in a pregnant woman, a surgeon with knowledge of the vascular anatomy of the upper abdomen should be immediately consulted. Rapid and multidisciplinary surgical management improves the patient's chance of survival [6]. In the event of a maternal death, an attempt can be made at salvage of the fetus via postmortem cesarean delivery if resuscitative efforts are unsuccessful after four minutes of cardiopulmonary resuscitation [6]. In cases where intraperitoneal hemorrhage is suspected preoperatively, the exploratory laparotomy is probably best accomplished through a vertical midline incision. Such an incision is amenable to extension in the cephalad direction to allow access to the upper abdomen.

The choice of treatment depends on the location of the SAA. Approximately, 80% of SAA are located in the distal portion of the splenic artery [5]. If the aneurysm is located in the proximal segment, it should be treated with simple ligation without arterial reconstruction since the short gastric vessels provide sufficient collateral blood flow to the spleen. Proximal and distal ligation can be attempted when the aneurysm occurs in the middle third of the splenic artery [2]. In these two cases, splenectomy should largely be avoided in order to preserve immune function [3, 5]. When the aneurysm is located in the distal third or in the splenic hilum, the aneurysm should be resected and a splenectomy should be performed [2, 6].

Radiologic screening of asymptomatic women who are pregnant or are planning pregnancy is not a pragmatic strategy due to its low prevalence in the general population. However, screening women for SAA who have multiple risk factors for asymptomatic SAA, such as a pregnant woman with liver disease, may be considered [3, 7]. While the majority of SAA are identified following rupture, others are identified incidentally on abdominal imaging. If the SAA is identified incidentally in a pregnant woman or a woman of childbearing age, a proactive approach in management should be taken. The current recommendation is for elective treatment of aneurysms >2 cm in maximal diameter in women of childbearing age [2]. However, it has been recently shown that up to half of splenic artery aneurysms that rupture during pregnancy are less than 2 centimeters in diameter. Due to the high fetal and maternal mortality associated with rupture during pregnancy, some experts recommend that all SAA, regardless of size, should be treated during pregnancy or in women of childbearing age [2, 5].

The rupture of a SAA during pregnancy is a rare event, but when it does occur, it is often associated with a high rate of maternal and fetal morbidity and mortality. Obstetricians and other emergency providers should consider a ruptured SAA in any pregnant woman who presents with an acute surgical abdomen. Prompt recognition and emergent laparotomy along with the availability of general or vascular surgery consultants are paramount to both maternal and fetal survival. In the rare minority of women of childbearing age who are discovered to have an asymptomatic SAA prior to rupture, a proactive approach to management should be undertaken due to the high risk of rupture in pregnancy.
